# A multinational, phase 2, randomised, adaptive protocol to evaluate immunogenicity and reactogenicity of different COVID-19 vaccines in adults ≥75 already vaccinated against SARS-CoV-2 (EU-COVAT-1-AGED): a trial conducted within the VACCELERATE network

**DOI:** 10.1186/s13063-022-06791-y

**Published:** 2022-10-08

**Authors:** Julia M. Neuhann, Jannik Stemler, Antonio Carcas, Jesús Frías-Iniesta, Ullrich Bethe, Sarah Heringer, Lea Tischmann, Marouan Zarrouk, Arnd Cüppers, Franz König, Martin Posch, Oliver A. Cornely

**Affiliations:** 1 Faculty of Medicine and University Hospital Cologne, Translational Research, Cologne Excellence Cluster on Cellular Stress Responses in Aging-Associated Diseases (CECAD), University of Cologne, Herderstr. 52, 50931 Cologne, Germany; 2 Faculty of Medicine, and University Hospital Cologne, Department I of Internal Medicine, Center for Integrated Oncology Aachen Bonn Cologne Duesseldorf (CIO ABCD) and Excellence Center for Medical Mycology (ECMM), University of Cologne, Kerpener Str. 62, 50937 Cologne, Germany; 3 German Centre for Infection Research (DZIF), Partner Site Bonn-Cologne Department, Herderstr. 52, 50931 Cologne, Germany; 4 Faculty of Medicine, Hospital La Paz, Clinical Pharmacology Service. Institute for Health Research (IdiPAZ), Universidad Autónoma de Madrid, Madrid, Spain; 5 Faculty of Medicine, Clinical Trials Centre Cologne (ZKS Köln), University of Cologne, Gleueler Str. 269, 50935 Cologne, Germany; 6 Center for Medical Statistics, Informatics, and Intelligent Systems Medical University of Vienna, Spitalgasse 23, 1090 Vienna, Austria

**Keywords:** SARS-CoV-2, COVID-19 vaccination, BNT162b2 (Comirnaty®^)^, mRNA-1273 (Spikevax®), Phase II, Randomisation, Advanced age, ≥75 years, Immunosenescence, Fourth dose, Booster, Variants of concern, VOC

## Abstract

**Background:**

In the ongoing COVID-19 pandemic, advanced age is a risk factor for a severe clinical course of SARS-CoV-2 infection. Thus, older people may benefit in particular from booster doses with potent vaccines and research should focus on optimal vaccination schedules. In addition to each individual’s medical history, immunosenescence warrants further research in this population. This study investigates vaccine-induced immune response over 1 year.

**Methods/design:**

EU-COVAT-1-AGED is a randomised controlled, adaptive, multicentre phase II protocol evaluating different booster strategies in individuals aged ≥75 years (*n*=600) already vaccinated against SARS-CoV-2. The initial protocol foresaw a 3rd vaccination (1st booster) as study intervention. The present modified Part B of this trial foresees testing of mRNA-1273 (Spikevax®) vs. BNT162b2 (Comirnaty®) as 4th vaccination dose (2nd booster) for comparative assessment of their immunogenicity and safety against SARS-CoV-2 wild-type and variants. The primary endpoint of the trial is to assess the rate of 2-fold antibody titre increase 14 days after vaccination measured by quantitative enzyme-linked immunosorbent assay (Anti-RBD-ELISA) against wild-type virus. Secondary endpoints include the changes in neutralising antibody titres (Virus Neutralisation Assay) against wild-type as well as against Variants of Concern (VOC) at 14 days and up to 12 months. T cell response measured by qPCR will be performed in subgroups at 14 days as exploratory endpoint. Biobanking samples are being collected for neutralising antibody titres against potential future VOC. Furthermore, potential correlates between humoral immune response, T cell response and neutralising capacity will be assessed.

*The primary endpoint analysis* will be triggered as soon as for all patients the primary endpoint (14 days after the 4th vaccination dose) has been observed.

**Discussion:**

The EU-COVAT-1-AGED trial Part B compares immunogenicity and safety of mRNA-1273 (Spikevax®) and BNT162b2 (Comirnaty®) as 4th SARS-CoV-2 vaccine dose in adults ≥75 years of age. The findings of this trial have the potential to optimise the COVID-19 vaccination strategy for this at-risk population**.**

**Trial registration:**

ClinicalTrials.govNCT05160766. Registered on 16 December 2021.

Protocol version: V06_0: 27 July 2022

**Supplementary Information:**

The online version contains supplementary material available at 10.1186/s13063-022-06791-y.

## Introduction

### Background

The novel coronavirus SARS-CoV-2 (severe acute respiratory syndrome coronavirus 2) was identified in December 2019 as a cause of severe pneumonia, acute respiratory distress syndrome (ARDS) and potential multiorgan failure in humans. The disease known as COVID-19 has since turned into a still ongoing pandemic affecting all countries in the world where it has caused widespread mortality and long-term morbidity.

A huge research effort on the prevention and treatment of COVID-19 has been ongoing globally. As of July 2022, over 8000 clinical trials related with COVID-19 have been registered in 150 countries of which over 700 are classified as vaccine studies [1, 2]. Vaccination against COVID-19 started on 27 December 2020 across the European Union. Efficacy and safety have been demonstrated for a number of vaccines, leading to currently six vaccines with marketing authorisation in Europe (as of July 2022) [3].

Clinical trials performed for marketing authorisation have demonstrated high efficacy preventing moderate and severe courses of COVID-19 disease.

However, there are few data on the efficacy of COVID-19 vaccines in special populations, particularly in individuals of advanced age [4–7]. A recent effectiveness study from Israel estimated the rate of confirmed infection and severe COVID-19 disease in individuals aged ≥60 who received 4th dose of BNT162b2. SARS-CoV-2 infection and severe COVID-19 were less frequent after a 4th dose than in recipients of only three doses of BNT162b2. Protection against confirmed Omicron variant infection appeared short-lived, whereas protection against severe illness did not wane during the study period [8–10].

This trial aims to evaluate the immune response of a fourth vaccine dose as a booster strategy against wild-type SARS-CoV-2 and its variants in individuals aged 75 and older already vaccinated against SARS-CoV-2. This investigation will likely provide useful information for vaccination programs in Europe and elsewhere.

EU-COVAT-1-AGED was initiated with a design that foresaw a 3rd vaccination in individuals 75 years of age or older (Part A). Evolving vaccination policies quickly adopted and recommended a 4th  vaccine dose for this population [2, 11]. Therefore, the design of the trial was amended after the enrolment of 53 subjects, to deliver 4th vaccination (2nd booster) as a study intervention from February 2022 onwards. This current design is referred to as Part B (*N*=550) of the trial and is presented in this paper.

Our study is conducted within VACCELERATE, the clinical research network for the coordination and conduct of COVID-19 vaccine trials in Europe. This EU-funded network comprises academic institutions all over Europe. The consortium is led by the University of Cologne, Germany, and currently includes 29 national partners in 18 EU member states and 5 countries associated to the EU Horizon 2020 research programme [12].

### Study rationale

Available data suggest that immunisation with any of the currently approved vaccines does not provide long-term or life-long protection [13]. Therefore, additional vaccinations, so-called booster doses, are indicated at some point after primary two-dose vaccination, especially so in the context of immunosenescence and with regard to emerging VOC of SARS-CoV-2 [6, 14]. Booster immunisations will play a crucial role to substantially improve the immune response against emerging VOC especially the Omicron lineage B.1.1.529 [15].

Omicron variants are causing a high infection incidence worldwide. Currently available monoclonal antibodies show impaired neutralising activity against predominant Omicron variants, limiting treatment options. A recent study has shown a remarkable increase in serum neutralising activity 1 month post a 3rd dose of BNT162b2. Titres were nearly similar to those observed against Wu01 after 2nd vaccination, booster vaccinations with available vaccines seem to be the most effective step in the prevention of infection [15]. With regard to the Omicron variant, future vaccination intervals may be kept close together timewise [13, 16].

In advanced age, the immune response is reduced and therefore shorter intervals between booster doses may be warranted for protection against SARS-CoV-2 infection [17]. There are no data on the ideal timing of booster doses in immunosenescent subjects. Also, in the age group ≥75 years no robust evidence has been published on the immune response elicited by heterologous booster strategies.

Therefore, EU-COVAT-1-AGED assesses the immunogenicity of different mRNA-based booster schedules in subjects ≥75 years and may aid in improving vaccination schedules in this specific high-risk population.

### Selection and justification of IMP dosage

*Please note*: The above summarizes the state of knowledge and assumptions at the time of the development of the trial design in late 2021.

mRNA-1273 will be used at a dose of 100 μg for all on-study vaccinations.

The European Medicine Agency (EMA) fully approved mRNA-1273 with a primary series of 2 vaccinations 28 days apart, each at a dose of 100 μg (0.5 mL) for individuals 12 years of age or older [18]. Additional vaccinations may be administered. A 3rd dose of 100 μg containing mRNA-1273 vaccine is approved for individuals with certain immunocompromising conditions 12 years of age or older [18]. In immunocompetent subjects at least 18 years of age or older, a single booster dose of mRNA containing 50 μg is approved by the EMA [18].

The US Food and Drug Administration (FDA) announced emergency use authorisation on 29th March 2022 for a 2nd booster dose (4th dose) at the 50 μg dose for adults over 50 years of age and adults over 18 years of age with certain kinds of immunocompromising conditions [19].

Initially, the rationale for selection of a reduced booster dose of 50 μg (0.25 mL) was provided by the manufacturer as follows:

- “Goal was to use optimal effective dose for boosting.
- Lower booster doses than those used for primary series of other vaccines [was] shown to reactivate immune memory.
- Lower booster dose increases worldwide vaccine supply of mRNA-1273” [20].

The decision for a reduced dose of the mRNA-1273 booster vaccine was not driven by safety concerns and was discussed before the Omicron variant became predominant. The Omicron variant, however, displays significantly higher transmissibility compared to the Delta variant [21] and evades vaccine-induced [22] as well as infection-induced immunity [23] to a greater extent compared to other variants. Higher concentration of neutralising antibodies is needed to prevent COVID-19 caused by the Omicron variant and its sub-variants. It was demonstrated that a 100 μg booster of mRNA-1273 resulted in a 2.5-fold increase of Omicron-specific neutralising antibodies compared to the 50 μg booster [24]. The higher concentration of neutralising antibodies will presumably lead to a better protection against severe COVID-19**.** The initial and approved dose of 30 μg is intended for vaccination with BNT162b2.

### Objectives

#### Primary objective

To compare the immune response between treatment arms after a 4th vaccination dose against SARS-CoV-2.

#### Safety objectives

To assess the safety of a 4th vaccination dose against SARS-CoV-2 in the study population.

#### Secondary objectives

To compare the humoral response against wild-type SARS-CoV-2 between treatment arms after a 4th vaccination dose against SARS-CoV-2.

To evaluate descriptively humoral response against SARS-CoV-2 variants of concern between treatment arms after a 4th vaccination dose against SARS-CoV-2.

To evaluate descriptively the long-term humoral immune response of 4th vaccination dose against SARS-CoV-2.

#### Exploratory objectives

To investigate the cellular immune response after a 4th vaccination dose, virus neutralising capacity against newly emerging variants in bio-banked samples and correlates of interest.

### Endpoints

#### Primary endpoint

- Rate of 2-fold antibody titre increase 14 days after a 4th vaccination dose measured by quantitative enzyme-linked immunosorbent assay (Anti-RBD-ELISA) against wild-type virus (Part B).

#### Safety endpoints

- Unsolicited AEs until the end of the trial.
- Solicited AEs for 7 days after a 4th vaccination dose.
- Rate of serious adverse events (SAEs) Grade ≥3 according to the National Cancer Institute Common Toxicity Criteria up to three months after a 4th vaccination dose.

#### Secondary endpoints

- Change in neutralising antibody titre (Virus Neutralisation Assay = VNA) against wild-type 14 days after a 4th vaccination dose, to be determined in a subgroup only.
- Change in neutralising antibody titre (VNA) against variants of concern 14 days after a 4th vaccination dose, to be determined in a subgroup only.
- Antibody titre level at 12 months after a 4th vaccination dose measured by a quantitative enzyme-linked immunosorbent assay (Anti-RBD-ELISA assay).
- Neutralising antibody titre (VNA) against wild-type SARS-CoV-2 at 12 months after a 4th vaccination dose, to be determined in a subgroup only.
- Neutralising antibody titre (VNA) against variants of concern at 12 months after a 4th vaccination dose, to be determined in a subgroup only.

#### Exploratory endpoints

- Change in cellular immune response (CD4+ and CD8+ T cell response) measured by qPCR 14 days after 4th booster dose, to be determined in a subgroup only.
- Neutralising antibody titre (VNA) against newly emerging variants in bio-banked samples after 4th vaccination dose, to be determined in a subgroup only.
- Correlates of the humoral immune response, cellular immune response, and viral neutralising capacity against SARS-CoV-2 variants of concern (VOC), to be determined in a subgroup only.

Please note that the size of the above-mentioned subgroups is targeted to be 200 subjects each. Analysis will be performed in all samples if additional funding becomes available.

### Trial design

**Fig. 1:**
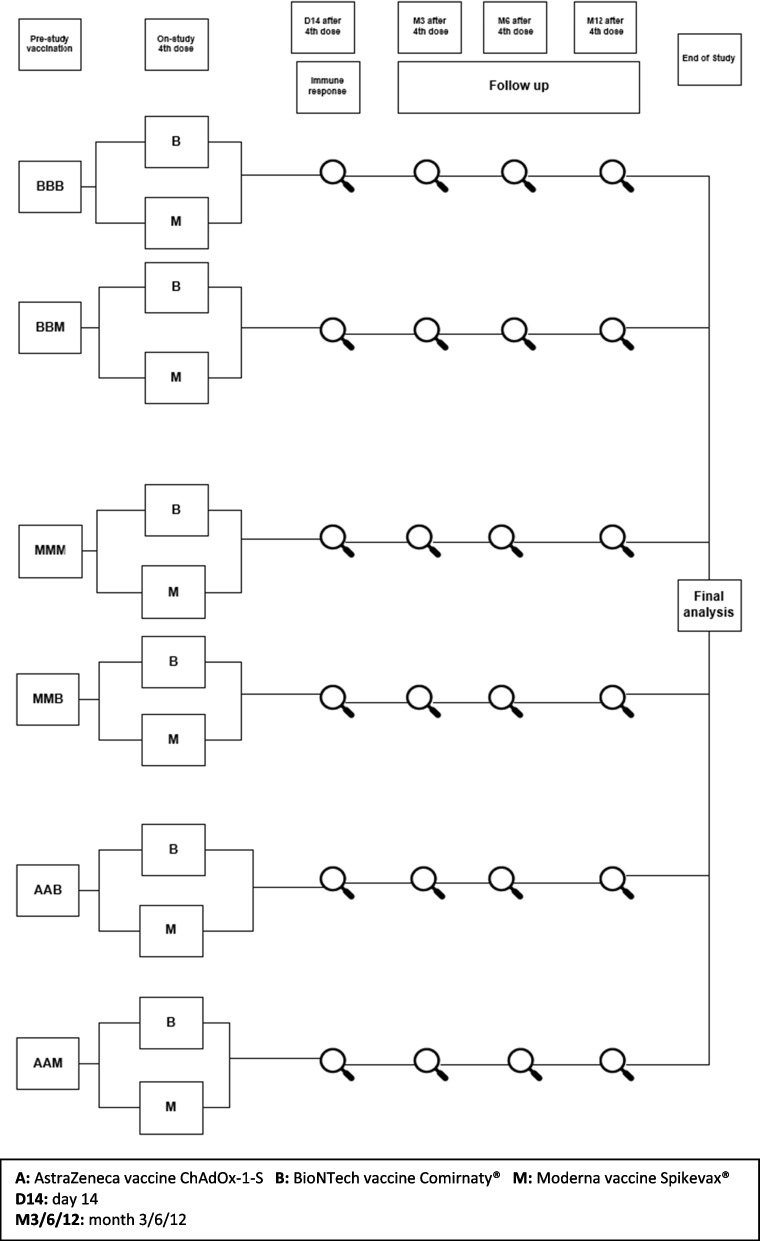
Flow chart of the trial design EU-COVAT-1-AGED Part B

This is a randomised controlled, adaptive, multicentre Phase II protocol evaluating the immunogenicity and reactogenicity of different strategies of a 3rd (Part A, not presented in this paper) and 4th (Part B) vaccination dose (“boosters”) in individuals ≥75 years after a primary vaccination series against SARS-CoV-2. Part B of this trial foresees testing of different vaccines as a 4th vaccination dose (also referred to as second booster vaccination) for comparative assessment of their immunogenicity and safety against SARS-CoV-2 wild-type and variants in individuals ≥75 years of age (see Fig. 1). The trial design follows a template from a master protocol developed within the VACCELERATE network.

Subjects in each cohort will be randomised to one of the arms planned in an equal allocation ratio. In each cohort, the number of arms foreseen at this moment is 2; therefore, the allocation ratio in each cohort will be 1:1.

For the flow chart of the trial design please refer to Fig. 1.

## Methods

### Participants, interventions, and outcomes

#### Study setting

This clinical trial will be conducted as a multicentre trial in up to 16 sites in up to 5 countries in Europe (approx. 3 sites per country), which are listed on ClinicalTrials.gov NCT05160766.

#### Trial sites

Centres selected will be validated by the VACCELERATE network according to approved standardised validation procedures.

All principal investigators and trial sites participating in this trial are required to have previous experience with the conduct of clinical trials with vaccines, to have experience in the regulatory process of their respective country, adequate facilities, sufficient dedicated and trained staff, access to target population and COVID-19 testing available on site.

All trial sites will be trained during the initiation visit by the clinical research associates (CRA).

#### Eligibility criteria

- Subject is ≥75 years old.
- For study entry in Part B the subject was vaccinated with one of the following vaccination regimens (1st + 2nd + 3rd dose):
  - BNT162b2 + BNT162b2 + BNT162b2
  - BNT162b2 + BNT162b2 + mRNA-1273
  - mRNA-1273 + mRNA-1273 + mRNA-1273
  - mRNA-1273 + mRNA-1273 + BNT162b2
  - ChAdOx-1-S + ChAdOx-1-S + BNT162b2
  - ChAdOx-1-S + ChAdOx-1-S + mRNA-1273
- The last dose of the above vaccinations must have been administered at least 1 month prior to study entry. Vaccination status should be documented in the source data and will be captured in the eCRF.
- No contra-indication against any of the vaccine products in the trial.
- Written informed consent from the subject has been obtained.

#### Exclusion criteria

- Prior to study entry, the subject got vaccinated with a regimen not included in the above list.
- Last anti-SARS-CoV-2 vaccine dose administered less than 1 month prior to study entry.
- Vaccination against a disease other than COVID-19 within 2 weeks prior to study entry. Only exception: Influenza vaccination which is allowed at any time.
- Subjects with any significant or uncontrolled disease posing a risk due to vaccination as judged by the investigator.
- Current immunosuppressive therapy, for example, continuous glucocorticosteroid treatment equivalent to >10 mg/day of prednisolone.
- Subject simultaneously participates in another clinical trial or has participated in the past 30 days.
- Subjects unable to report solicited adverse events.
- Subject participates or participated in Part A of this trial.
- Subject with any contraindications to the vaccines in the trial. A list of contraindications as listed in the Summary of medicinal Product Characteristics (SmPC, the Fachinformation in Germany), if appropriate.
- Use of drugs with significant interaction with the investigational product according to the SmPC or similar documents.
- Diseases or findings that may have a significant effect on the target variables and which may therefore mask or inhibit the therapeutic effect under investigation.
- Subject had COVID-19 or tested positive for SARS-CoV-2 within the last 3 months.
- Persons with any kind of dependency on the principal investigator or employed by the sponsor or principal investigator.
- Legally incapacitated persons.
- Persons held in an institution by legal or official order.

### Interventions

Study vaccines will be given as a 4th dose in subjects already vaccinated with defined prior vaccination strategies (with BNT162b2, mRNA-1271 or ChAdOx-1-S) as per eligibility criteria. The vaccines to be evaluated as 4th vaccination (second booster) dose will be BNT162b2 (Comirnaty®) and mRNA-1273 (Spikevax®). For a detailed overview of all cohorts and arms in this study please refer to Table 1.

**Table 1:** EU-COVAT-1-AGED intervention in Part B – 4th vaccination dose^b^

Cohort	Vaccination prior to study entry	Arm	Study intervention: 4th vaccination dose^**a**^
Cohort 4	BNT162b2 + BNT162b2 + BNT162b2	7	BNT162b2
		8	mRNA-1273
Cohort 5	BNT162b2 + BNT162b2 + mRNA-1273	9	BNT162b2
		10	mRNA-1273
Cohort 6	mRNA-1273 + mRNA-1273 + mRNA-1273	11	BNT162b2
		12	mRNA-1273
Cohort 7	mRNA-1273 + mRNA-1273 + BNT162b2	13	BNT162b2
		14	mRNA-1273
Cohort 8	ChAdOx-1-S + ChAdOx-1-S + BNT162b2	15	BNT162b2
		16	mRNA-1273
Cohort 9	ChAdOx-1-S + ChAdOx-1-S + mRNA-1273	17	BNT162b2
		18	mRNA-1273

^a^Administered at least 1 month after the 3rd pre-study vaccination

^b^For intervention in Part A (3rd vaccination dose) see Additional file 1

In Part B there is a minimum interval of 1 month between the on-study 4th vaccination dose and the 3rd pre-study vaccination, with longer intervals as per local regulation or practice.

#### Study visits and assessments

For a detailed overview of all study visits and assessments please refer to Table 2.

**Table 2:** EU-COVAT-1-AGED – Visit Schedule Part B

Visit number	1	2	3	4	5
Procedure	Screening, enrolment, baseline 4th dose	Immune response evaluation	Follow-up	Follow-up	End of study
Day ± window	0	14 ±2 days after 4th dose	3 months ± 3 days after 4th dose	6 months ± 3 days after 4th dose	12 months ± 3 days after 4th dose
**Screening for eligibility**					
Informed consent^a^	X				
Demographics and medical history^f^	X				
Eligibility check	X				
**Baseline procedures**					
Concomitant medication review	X	X			
Physical exam	X	X^b^			
Vital signs	X	X^b^			
**Immunogenicity**					
Anti-RBD and anti-N IgG (ELISA)	X	X			X
Neutralising activity (wild-type)^c^	X	X			X
Neutralising activity (VOC)^c^	X	X			X
Cellular response (qPCR assay)^c^	X	X			
Biobanking^d^	X	X	X	X	X
**IMP administration**					
Vaccination^g^	X				
**Safety**					
AE/SAE^e^	X	X	X	X	X

*AE* adverse events, *SAE* serious adverse event

^a^Informed consent must be obtained before obtaining consent for biobanking and secondary data use, and any other procedure to be undertaken.

^b^Will be performed at visit 2 only upon SAE

^c^Samples taken from all subjects, analysis performed in a subgroup only. Analysis will be performed in all samples if additional funding becomes available.

^d^For secondary use defined in informed consent; also optional at visit 1 and visit 2: at trial site and upon agreement of trial participant additional blood collection for biobanking of peripheral blood mononuclear cells (PBMC) as per informed consent

^e^Solicited AEs are recorded by trial participant till Day 7 and records will be collected at visit 2 (Day 14) by trial staff and captured in eCRF; unsolicited AEs are recorded by trial investigator until the end of trial as described in this protocol

^f^Medical history includes information on prior SARS-CoV-2 infection and COVID-19 disease if applicable; name of SARS-CoV-2 variant should be documented if known

^g^Administration of 4th vaccination dose after blood sampling for immunogenicity and cellular immunity during visit 1. Trial participant is observed for any adverse reaction for at least 15 min or according to standard of care upon vaccination

#### Withdrawal

A participant may withdraw from the study at any time at his/her own request or may be withdrawn at any time at the discretion of the investigator for safety, behavioural, or compliance/adherence reasons. This is expected to be uncommon. When a participant withdraws before study completion, the reason for withdrawal is to be documented in the eCRF and in the source document. If the reason for withdrawal from the study is withdrawal of consent, then no additional assessments are allowed. The participant will be permanently discontinued from the study intervention and the study at that time. If the participant withdraws consent for disclosure of future information, the sponsor may retain and continue to use any data collected before such a withdrawal of consent. If a participant withdraws from the study, he/she may request destruction of any samples taken and not tested, and the investigator must document this in the study site records.

### Outcomes

#### Outcome measures and efficacy assessment

Assessments include the primary and secondary endpoints described above. The choice of these outcome parameters is in line with practice in other COVID-19 vaccination trials. The primary endpoint in particular was discussed and agreed upon with the European Medicine Agency when the initial trial design was developed.

All data will be captured in eCRF. The primary endpoint is measured by means of quantitative enzyme-linked immunosorbent assay (Anti-RBD-ELISA) against wild-type virus at day 14 following on-trial vaccination.

Occurrence of AEs and serious SAEs during the trial will be also documented in the eCRF.

### Overall study timeline

Proposed overall timeline (Table 3)

**Table 3:** EU-COVAT-1-AGED – time schedule

First patient first visit (FPFV)	November 2021
Last patient first visit (LPFV)	September 2022
Last patient last visit (LPLV)	September 2023
Analysis	October 2022 (Primary endpoint analysis)
End of study definition	The end of study will be on the day of database lock.
End of trial	November 2023
Final study report	December 2023

### Assignment of interventions

#### Patient randomisation

Randomisation will be implemented by a 24/7-Online service (ALEA 17.1, FormsVision BV, Abcoude, NL) and prepared centrally by the Institute of Medical Statistics and Computational Biology (IMSB) at the University of Cologne.

Subjects fulfilling selection criteria will be randomised to 4th dose through a central procedure. Subjects will be randomised in a 1:1 ratio to one of the two treatment groups:

- 4th vaccination with BTN162b2 (Comirnaty®), including modified vaccine product.
- 4th vaccination with mRNA-1273 (Spikevax®), including modified vaccine product.

Stratification is planned according to the pre-study vaccination series eligible for Part B (primary vaccination plus 3rd vaccine dose (= first booster), gender (female/male), documented history of prior COVID-19 infection (yes/no), as applicable and defined also by enrolment criteria.

#### Blinding

The design of the trial is open label so blinding will not occur.

### Data collection, data management, and data analyses

The IT infrastructure and data management are provided by the Clinical Trials Centre Cologne (CTCC).

The trial database is developed and validated by the CTCC on the basis of standard operating procedures. All changes made to the data will be documented in an audit trail. The commercial online software TrialMaster^TM^ (Anjusoftware.com) is used as a data management system, ensuring data safety by a firewall and backup system, including multiple data storage sites. The data are backed up daily.

All relevant trial data collected during the trial are documented soon after data gaining and entered into the eCRF by the responsible investigators or a person authorised by the principal investigator.

This includes all outcome measures and questionnaires. Automatic plausibility checks are run during data entry to immediately detect discrepancies. Data management of the CTCC carries out checks on the completeness and plausibility of the study data and clarifies all queries with the study centre electronically via the trial software. These queries must be answered promptly by the study centre. The eCRFs are signed by the principal investigator at the end of a patient's trial participation to confirm the accuracy of the data. After completion and cleaning of the data, the database is closed, and the data will be exported for statistical analysis.

To monitor risks and ensure the data quality of the trial, a central quality control process (cQC) was set up.

Using cQC, the performance of the trial sites indicating the documentation rate, documentation quality and recruitment rate will be evaluated. In the case of conspicuous data (e.g. incorrect, or insufficient documentation), this quality control process allows for early intervention and correction of errors.

### Data protection

The provisions of the EU General Data Protection Regulation 2016/679 (GDPR) are observed. It is ensured that all investigational materials and data are pseudonymised in accordance with data protection regulations before scientific processing.

Subjects will be informed that their pseudonymised data will be passed on in accordance with applicable regulations to the recipients described there. Subjects, who do not agree to the data handling as described in the informed consent, will not be included in the trial. The investigator or sub-investigator (SI) will obtain the informed consent.

### Statistical methods

#### Sample size calculation

The number needed to achieve a certain precision to estimate the primary endpoint has been calculated at 550 for part B (i.e. 600 overall for Part A and B, including a potential dropout rate of 8–10% for Part B).

#### Sample size calculation with multiplicity adjustment

Sample size calculation has been carried out for the primary endpoint for the rate π of 2-fold antibody titre increase following 4th dose vaccination. As two-sided simultaneous 95% confidence intervals for this rate should be calculated separately for each randomised group in Part B a Bonferroni adjustment was used in the sample size calculation accordingly.

When the sample size is 250 per randomised group, two-sided simultaneous 95% confidence intervals (with Bonferroni adjustment for 2 simultaneous confidence intervals in a cohort) for a proportion using the large sample normal approximation will extend no more than ±7.1% (percentage points) from the observed proportion, e.g. if the observed proportion is 50% (where the confidence interval is widest), the confidence interval ranges from about 42.9% to 57.1%.

To adjust for potential dropouts in Part B of about 8-10%, the total sample size for Part B (=4th vaccination and cohorts 4–9) was set to 550.

In Table 4, the precision for the simultaneous two-sided 95% confidence intervals (with the same assumptions as described above) is shown for varying sample sizes. This shows which precision could be achieved in the different cohorts based on the previous vaccination strategy (see also inclusion criteria for Part B).

**Table 4:** EU-COVAT-1-AGED sample size calculation

**Sample size,** ***n***	250	200	150	125	90	50	40
**Expected proportion, π**	0.500	0.500	0.500	0.500	0.5	0.500	0,5
**Distance from proportion to Limit, ω**	0.071	0.079	0.092	0.100	0.118	0.158	0.177

### Software programs

All statistical analyses will be conducted with statistical software like SAS 9.4. or higher and R 3.6.3. or higher.

All *primary analyses* will be on the mITT set. The primary endpoint is the rate π of 2-fold antibody titre increase following the 4th vaccine dose measured by quantitative enzyme-linked immunosorbent assay (Anti-RBD-ELISA) against wild-type virus at 14 days after the 4th vaccine dose (versus immediately before vaccination).

For the binary primary endpoint absolute and frequencies in per cent will be calculated for each treatment group. For each study part and treatment group, the corresponding rate together with simultaneous 95% confidence intervals will be calculated. Exact Clopper Pearson confidence intervals at Bonferroni adjusted level of (1–0.05/2) *100% will be provided.

In additional supportive analysis, the primary endpoint will be performed in the same way for each cohort separately, i.e. further exploratory analyses will be performed based on different pre-vaccination series. Especially, within each cohort, a multiplicity adjustment will be implemented

The data will be visualised with bar charts.

For the second part B (4th vaccination, cohorts 4–9), the primary endpoint analysis will be triggered as soon as for all patients the primary endpoint data (14 days after study vaccination) has been captured.

### Secondary analyses

The geometric mean titres (GMT) following, and the 4th dose vaccination measured by quantitative enzyme-linked immunosorbent assay (Anti-RBD-ELISA) against wild-type virus at 14 days after the 4th dose will be compared between the boosters BNT162b2 and mRNA-1273 computing two-sided 95% confidence intervals for the GMR or differences of GMC on log scale (base 10). Based on the confidence intervals, an equivalence test will be performed. The equivalence margins are the 1.5 to 0.67-fold change between the GMT in the two boost arms corresponding to a margin for the differences of GMC on log scale (base 10) which results in a margin ±0.174. This is suggested in Section 2.3.1 of the EMA/117973/2021 Reflection paper on the regulatory requirements for vaccines intended to provide protection against variant strain(s) of SARS-CoV-2. Because GMT are expected to have a skewed distribution, the log10 scaled GMT values will be compared using a linear model with factors booster (BNT162b2/mRNA-1273) as well as the stratification variables used in the randomisation. As this is a secondary analysis, no adjustment for multiplicity will be made. For Part B in further secondary analyses, it will be explored whether switching or keeping the same vaccination for the 4th vaccination (study intervention in B) compared to the 3rd vaccination will have an effect. In this model, the interaction between cohort and study drug will be included as well.

### Exploratory Analyses

Different combinations of vaccines (1st, 2nd, 3rd and the on-study 4th vaccine dose) will be assessed for equivalence in terms of humoral and/or cellular immune response against VOC compared to approved homologous COVID-19 vaccination. Furthermore, exploratory comparisons of the primary endpoint as well as the humoral and/or cellular immune response against VOC will be performed between cohorts as well as between the treatment groups within cohorts. As an option only in Part B, it is planned to run comparisons with the control group from EU-COVAT subprotocol with the EudraCT no.: 2021-004889-35. The control group of EU-COVAT subprotocol EudraCT no. 2021-004889-35 will be only utilised if the sample size in the age group of interest is sufficiently large. Otherwise, the data of the control group will be reported only descriptively. These analyses will be performed similarly to the analysis for the secondary analyses.

### Safety analyses

Summaries and analysis of safety data will be presented for the safety analysis population.

An adverse event (AE) is any untoward medical occurrence in a trial subject administered an IMP. There does not necessarily have to be a causal relationship with this treatment. The AE may be, but is not restricted to: a new illness, worsening of a sign or symptom following trial-specific treatment (i.e. 4th vaccination dose), the clinically significant abnormal results of an examination (e.g. laboratory findings, electrocardiogram) or deterioration of a pre-existing medical condition, or a combination of two or more of these factors, as applicable.

AEs will be documented starting from baseline visit and until the last scheduled visit after the 4th vaccine dose unless otherwise defined in the sub-protocol-specific visit schedule.

The safety endpoints include

- Unsolicited AEs until the end of the trial.
- Solicited AEs for 7 days after a 4th vaccination dose.
- Rate of serious adverse events (SAEs) Grade ≥3 according to the National Cancer Institute Common Toxicity Criteria up to three months after a 4th vaccination dose.

Listing will be provided on a subject level reporting the severity and relationship to study vaccination (related/unrelated). Absolute and numbers in per cent will be given per intervention group. Different types of AEs will be grouped. Additionally, the safety data will be reported descriptively for each intervention and cohort separately.

### Multiplicity adjustment

The different pre-study vaccine combinations are considered as inferentially independent, as the prior vaccination strategy cannot be influenced and there will be no extrapolation from one cohort to another one for the primary analysis. Therefore, the multiplicity adjustment concerning the primary endpoint will be applied within each cohort, but there will be no further adjustment for having several cohorts within the accompanying master protocol.

### Subgroup analyses

Subgroup analyses will be performed by gender and documented history of prior COVID-19 infection (yes/no), respectively.

### Interim analyses

An interim look will be performed as soon as 50% of participants have been recruited within Part B. In the interim analysis, Haybittle–Peto boundaries of 0.0001 will be used to calculate the confidence intervals for the primary analysis in each cohort. There will be not stopping because of differences in the primary endpoint analysis at interim analysis.

If a treatment arm is dropped due to safety concerns, a sample size re-allocation to the remaining arms might be considered. However, in this case, the overall sample is still fixed as initially planned, i.e. the total sample size over all cohorts in Part B is bounded by 550 patients in total.

The interim analysis will allow for a sample size reassessment in the interim analysis, e.g. to re-estimate the standard deviation. If a sample size reassessment is performed, the conditional error principle is implemented to control the FWER for the primary endpoint.

Note: In case enrolment is impacted by the dynamics of the pandemic and/or by public vaccination policies much faster than assumed at the time of trial design, the interim analysis may be omitted.

### Monitoring

The exact extent of the monitoring procedures is described in a separate monitoring manual. The Monitoring is performed risk based. Each trial site was subjected to a site selection visit to ensure staff qualification and experience, and sufficient capacity and equipment. The initial training of each site was performed during an initiation visit prior to site activation.

The trial sites will be closely monitored by a CRA ensuring the data’s quality, the trial subject’s safety and rights as a study participant. The monitor will check if accurate, valid and complete data are collected, and whether the trial is conducted in accordance with the trial protocol, the principles of GCP and local legislation. A close-out visit will be performed on each trial site for formal termination of the trial at the respective trial site.

All principal investigators agree that the monitor regularly visits the trial site and assure that the monitor will receive appropriate support in their activities at the trial site, as agreed in separate contracts with each trial site. The declaration of informed consent includes a statement allowing the monitor to compare the electronic case report form (eCRF) with the trial subject’s medical records (doctor’s notes, ECGs, laboratory printouts etc.).

The principal investigator will secure access for the monitor to all necessary documentation for trial-related monitoring. The aims of the monitoring visits are as follows:

- To check the correct obtaining process of informed consent forms.
- To monitor trial subject safety (occurrence and documentation/reporting of AEs and SAEs).
- To check the completeness and accuracy of entries on the eCRF
- To validate the entries on the eCRF against those in the source documents (source data verification (SDV)).
- To perform drug accountability checks.
- To evaluate the progress of the trial.
- To evaluate compliance with the trial protocol.
- To assess whether the trial is being performed according to GCP at the trial site.
- To discuss with the principal investigator aspects of trial conduct and any identified deficiencies.

A monitoring visit report is prepared for each visit describing the progress of the clinical trial and any problems. The principal investigator will reasonably consider the corrective and preventive measures suggested by the monitor. All patient data relating to the study will be recorded on an electronic CRF. The investigator is responsible for verifying that data entries are accurate and correct by electronically signing the eCRF. Guidance on completion of eCRF will be provided in the Trial Master File. The investigator must permit study-related monitoring, audits, REC/NCA review, and regulatory agency inspections and provide direct access to source data documents.

Monitoring details describing strategy, including definition of study critical data items and processes (e.g. risk-based initiatives in operations and quality such as risk management and mitigation strategies and analytical risk-based monitoring), methods, responsibilities, and requirements, including handling of noncompliance issues and monitoring techniques (central, remote, or on-site monitoring) are provided in the monitoring plan. The sponsor or designee is responsible for the data management of this study, including quality checking of the data. The sponsor assumes accountability for actions delegated to other individuals (e.g. contract research organisations).

Specific additional monitoring details for this trial are outlined in a separate monitoring plan.

CRA. Monitoring reports have to be written within 15 working days after the visit.

The original finalised monitoring report must be signed by clinical PM and then forwarded to the principal coordinating investigator (PCI, Leiter der klinischen Prüfung in Germany), who will countersign the report.

#### Data monitoring committee

An independent data monitoring committee (DMC) consisting of independent scientists not otherwise involved in the trial will be appointed and will review the data regularly during the study for safety and scientific integrity and will make recommendations to the sponsor regarding the stopping of an intervention for harm or for futility. The frequency of the DMC meetings and other aspects such as stopping rules will be described on an agreed charter. The DMC will be informed of all safety-relevant events by the sponsor.

#### Adverse events and safety reporting

All AEs and SAEs occurring during the trial will be documented in the patient’s medical records and the eCRF, including date and time of onset and resolution, severity, causal relationship with the on-trial vaccination, seriousness and measures. In addition, SAEs and AEs are immediately sent to the principal investigator (PI) and SIs via an automatic e-mail notification directly from the database. The risk-benefit ratio will be checked by the Investigator team based on subject’s medical history and a list of AEs that have occurred up to that point. The investigators will assess therefore every AE whether a causal relationship with the IMPs (BTN162b2 (Comirnaty®), mRNA-1273 (Spikevax®)) and/or study procedure can be assumed or not. All safety-relevant events will be promptly reported to the Ethics Committees, Sponsor and the DMC. The investigator will inform the sponsor of the occurrence or receipt of knowledge of the occurrence of an SAE without delay, at the latest within 24 h of being made aware of the event. The study specific SAE report forms must be completed and submitted by fax or e-mail to the CTCC to which the sponsor has delegated the SAE management procedures.

In the interest of participants’ safety, follow-up for up to 30 days after the individual participant’s study termination (individual study termination is defined as last visit) is required for SAEs that are not sufficiently resolved at the participant’s final trial visit, if applicable.

#### Auditing

As part of quality assurance, the sponsor has the right to audit the trial sites and any other institutions involved in the trial. The aim of an audit is to verify the validity, accuracy and completeness of data, to establish the credibility of the clinical trial, and to check whether the trial subject’s rights and trial subject safety are being maintained. The sponsor may assign these activities to persons otherwise not involved in the trial (auditors). These persons are allowed access to all trial documentation (especially the trial protocol, case report forms, trial subjects’ medical records, drug accountability documentation, and trial-related correspondence).

The sponsor and all trial sites involved undertake to support auditors and inspections by the competent authorities at all times and to allow the persons charged with these duties access to the necessary original documentation.

All persons conducting audits will keep all trial subject data and other trial data confidential.

### Ethics and dissemination

This clinical trial protocol received favourable ethics opinions and approval by national competent authorities in the following countries:

1. Germany, participating sites:

- University Hospital Cologne (01-01), Kerpener Straße 62, 50937 Cologne, Germany
- University Hospital Frankfurt (01-02), Theodor-Stern-Kai 7,60590 Frankfurt, Germany
- University Hospital Hannover (MHH) (01-03), Carl-Neuberg-Straße 1, 30625 Hannover, Germany
- Ethics Committee at the Medical Faculty of the University of Cologne: 28 September 2021, Ref: 21-1457_AMG-ff, 08^t^ November 2021 Ref.: 21-1457_2-AMG-ff, 29 November 2021 Ref.: 21-1457_3-AMG-ff & 21-1457_4-AMG-ff, 08 December 2021 Ref.: 21-1457_5-AMG-ff, 21 January 2022 Ref.: 21-1457_6-AMG-ff, 24 March 2022 Ref.: 21-1457_7-AMG-ff, 3 June 2022 Ref.: 21-1457_8-AMG-ff
- NCA: PEI: 20 October 2021 Ref.: 4647, PEI 1 December 2021 Ref.: 4647/02, PEI 21st January Ref.: 4647/03

2. Spain, participating sites:

- 05-01: La Paz University Hospital, Paseo de la Castellana 261, 28046 Madrid, Spain
- 05-02: BIODONOSTIA Health Research Institute (with registered office at PWith registered office at P0), Dr. Begiristain s/n, 20014 Donostia-San Sebastian (Gipuzkoa), Spain
- 05-03: Hospital De Bellvitge, Feixa Llarga, s/n, 8907, Hospitalet de Llobregat, Barcelona, Spain
- 05-04: Hospital Universitari Germans Trias i Pujol, Clinical Pharmacology Unit and Infectious Diseases Department, Carretera de Canyet, s/n, 08916 Badalona (Barcelona), Spain
- 05-05: CS Carlos Castilla del Pino, Avda. Menéndez Pidal s/n, 14004 Córdoba, Spain
- Ethics Committee of La Paz University Hospital: 11 March 2022 Ref.: 6101
- NCA Spanish Agency of Medicines and Health Products: 16 March 2022 Ref.: MUH/CLIN/EC

3. Norway, participating sites:

- 04-01: University Hospital Haukeland, Helse Bergen HF, Dept of Internal Medicine, Jonas Lies vei 65, 5021 Bergen, Norway
- REK - Regional Committees for Medical and Health Research Ethics: 25 May 2022
- NCA Norwegian Medicines Agency: 15 March 2022 Ref.: 22/02447-11

4. Lithuania, participating sites:

- 03-01: Inlita JSC, Santariskiu str. 5, LT08406, Vilnius, Lithuania
- 03-02: Vilnius University Hospital Santaros klinikos Centre of Infectious Diseases, Santariskiu street14, LT-08410 Vilnius, Lithuania
- Lithuanian Bioethics Committee: 17 May 2022 Ref.: 2022-05-31 No. P-22-19/2
- NCA State Medicines Control Agency under the Ministry of Health of the Republic of Lithuania: 24 May 2022 Ref.: Nr. (1.4E)1A-580

5. Ireland, participating sites:

- 02-01: Beaumont Hospital, Beaumont Road, Dublin 9, D09V2N0, Ireland
- 02-02: Cork University Hospital, Bishopstown Road Cork, Cork,County Cork, Ireland
- 02-03: Mater Misericordiae University Hospital, Eccles Street, Dublin 7. D07 R2WY. Ireland
- 02-04: St. James’s Hospital, James Street, Dublin 8. D08 NHY1. Ireland.
- 02-05: St. Vincent’s University Hospital, Elm Park Dublin 4. D04 T6F4. Ireland
- NREC - National Office for Research Ethics Committee: 24 June 2022 Ref.: 22-NREC-CT-091
- NCA HPRA - Health Products Regulatory Authority Ireland: 11 March 2022 Ref.: 2215807

After receiving favourable ethics opinion on 8 November 2021 (Ref.: 21-1457_2-AMG-ff) and approval by the Paul-Ehrlich-Institut (PEI) on 20 October 2021 (Ref.: 4647), enrolment for Part A of the study started in November with protocol version 03_0 in Germany at the trial site Cologne. For subsequent approvals, see the “Amendments” section.

The current version 06_0 of the clinical trial protocol was submitted to ethics bodies and national competent authorities in the participating countries in July 2022 (for details, see the “Amendments” section).

In case of important protocol modifications, the PCI will inform the PIs of all trial sites. They will then forward the amendments to the associated ethics committees and trial participants. Written informed consent is provided by all participants prior to any trial procedure. A subject insurance policy is taken out for all enrolled patients. Address, insurance number, telephone and fax number of the subject insurance company are included in the informed consent. In addition, subjects will be insured against travel accidents on their way to the trial site (only applies for trial sites in Germany).

The trial is conducted in accordance with the International Conference on Harmonization for Good Clinical Practice (ICH-GCP). The trial will comply with the Declaration of Helsinki at any time.

The results of this trial will be submitted for publication in peer-reviewed scientific journals and presented at national and international conferences.

Please Note: The VACCELERATE project is conducted under Article 48 of the Regulation (EC) No 536/2014 and the ICH guidelines on Good Clinical Practice. All research activities comply with ethical principles and applicable international, EU and national law, in accordance with Article 34 of the Horizon 2020 Model Grant Agreement.

### Amendments

Significant changes will be implemented after approval by the national competent authority and favourable opinion of the ethics committee, only.

The amendment from protocol version V04_0 to V05_0 terminated Part A of this study with a 3rd vaccination as study intervention and introduced Part B with a 2nd booster vaccination (i.e. 4th vaccination dose) (EC Ref.: 21-1457_6-AMG-ff, 21 January 2022; PEI Ref.: 4647/03¸ 21 January 2022).

The amendment from protocol version V05_0 to V06_0 foresaw clarifications and correction of editing errors without changing the trial design. This amendment was submitted to ethics bodies and national competent authorities in the participating countries on 27 July 2022.

## Discussion

The worldwide COVID-19 pandemic has had a profound impact on clinical trials, including the design and conduct of the present EU-COVAT-1-AGED trial Part B.

The continuous emergence of new VOC with high transmissibility and ability to escape immune response challenges prevention and treatment of COVID-19 in a variety of hosts, including those at particular risk [14, 17, 25–27].

Individuals of advanced age benefit from boosting immune response with additional mRNA vaccine doses [7, 8, 28]. Factors of natural immunosenescence and individual medical history alike, influence waning of immune response. To date, only few systematic trials exist for this specific population [6, 29, 30].

The multicentre, randomised EU-COVAT-1-AGED study was designed to compare immunogenicity and safety of mRNA-1273 and BNT162b2 booster doses from 14 days and up to 12 months. Since start of recruitment in November 2021 Part A of this study investigated immune response to a first booster (=3rd vaccination dose). Fifty-three subjects were enrolled at the trial site Cologne. In January 2022 the study had then to be redesigned and amended for 4th vaccination dose as study intervention reacting to the EU-wide change of vaccination policies including the German Robert-Koch-Institut (RKI) recommending the 4th vaccination for people ≥70 years in January 2022 [31].

The successful conduct of clinical vaccination trials is hampered by several obstacles as seen with this trial.

Continuously emerging VOC and their subvariants demand modification/development of for example new vaccines or concomitant therapies implicating time-consuming and expensive amendments containing the expansion of the trials IMPs by modified products, the increasing of dosing or the changing in the intervention’s timing [32].

On top, regulatory processes are time-consuming. Regulatory bodies should also intend to adapt to the rapid change in the pandemic. This situation has already forced a reassessment of clinical trial conduct and feasibility. Within the VACCELERATE platform, a pan-European network for improving and acceleration of vaccination trials, processes of designing, coordination and conducting are already harmonised [12, 33]. However, national competent authorities and different ethics committees in the participating countries often limit the simultaneous conduct and recruitment of matching subjects in time. Using EMA’s Clinical Trials Information System (CTIS) was no option at the time of the initial trial design. This multifactorial delay in the conduct of vaccination trials in this case prevents the optimal timing for data publishing, which in turn is a necessity for progress against the pandemic [34, 35].

The need for clinical trials optimising booster strategies seems obvious in light of the ongoing COVID-19 pandemic. We still face viral mutation and increased viral transmissibility resulting in considerable mortality and morbidity in vulnerable populations such as immunocompromised individuals and older people [15, 36].

This trial generates in-depth data on individual long-term immune response after a 4th vaccination dose in people aged ≥75 years, i.e. highly specific data not available so far. The results may contribute to optimise future vaccination schedules for this at-risk cohort.

## Trial status

The authors confirm that the trial was registered prior to the submission of this manuscript (ClinicalTrials.gov Identifier: NCT05160766, EudraCT Number: 2021-004526-29 registered on. 16th of December, 2021). Recruitment of participants in Part B started in February 2022; the last patient’s last visit is expected in autumn 2023.

At the time of submission of this manuscript, the trial had enrolled 263 subjects of 316 in Part B.

## Supplementary Information

**Additional file 1.** EU-COVAT-1-AGED Intervention in Part A – 3rd vaccination

**Additional file 2.**

## Data Availability

Any data required to support the protocol can be supplied on request.

## Abbreviations

AE: Adverse event
Anti-N: Anti-nucleocapsid antibody
CEMSIIS: Center for Medical Statistics, Informatics and Intelligent Systems (University of Vienna)
cQC: Central quality control
CRA: Clinical research associate
CTCC: Clinical Trials Centre Cologne (ZKS)
CTIS: Clinical Trials Information System
DMC: Data Monitoring Committee
EC: Ethics Committee
eCRF: Electronic case report form; GCP: Good Clinical Practice
GDPR: General Data Protection Regulation
ICH-GCP: International Conference on Harmonization for Good Clinical Practice
IdiPAZ: Hospital La Paz Institute for Health Research
IMP: Investigational Medical Product
NCA: National Competent Authorities
PBMC: Peripheral blood mononuclear cells
PI: Principal Investigator
PCI: Principal Coordinating Investigator
Ref.: Reference
RKI: Robert-Koch-Institut
SARS-CoV-2: Severe Acute Respiratory Syndrome Coronavirus 2
SAE: Serious adverse event
SI: Sub-Investigator
VNA: Virus Neutralisation Assay
VOC: Variants of concern
