# Supplementary material for: A multinational, phase 2, randomised, adaptive protocol to evaluate immunogenicity and reactogenicity of different COVID-19 vaccines in adults ≥75 already vaccinated against SARS-CoV-2 (EU-COVAT-1-AGED): a trial conducted within the VACCELERATE network
Source: Trials. 2022 Oct 8;23:865. doi: 10.1186/s13063-022-06791-y (PMC9547672; doi:10.1186/s13063-022-06791-y)
Supplement: Supplementary file 1 — Additional file 1. EU-COVAT-1-AGED Intervention in Part A – 3rd vaccination [file 13063_2022_6791_MOESM1_ESM.docx]

**Additional file 1**

**EU-COVAT-1-AGED Intervention in Part A – 3^rd^ vaccination**

| **Cohort** | **Vaccination prior to study entry** | **Arm** | **Study intervention:**  **3^rd^ vaccination dose** | **Part A**  **with Cohorts 1 to 3 closed to further recruitment as of January 13, 2022** |
| --- | --- | --- | --- | --- |
| Cohort 1 | BNT162b2  +  BNT162b2 | 1 | BNT162b2 |  |
|  |  | 2 | mRNA-1273 |  |
| Cohort 2 | mRNA-1273 +  mRNA-1273 | 3 | BNT162b2 |  |
|  |  | 4 | mRNA-1273 |  |
| Cohort 3 | ChAdOx-1-S +  ChAdOx-1-S | 5 | BNT162b2 |  |
|  |  | 6 | mRNA-1273 |  |
| Control | Control arm of the EU-COVAT subprotocol EudraCT no. 2021-004889-35, a separate sub-protocol embedded within the EU-COVAT master protocol, will be used for a descriptive comparison. | | |  |
