# Supplementary material for: A multinational, phase 2, randomised, adaptive protocol to evaluate immunogenicity and reactogenicity of different COVID-19 vaccines in adults ≥75 already vaccinated against SARS-CoV-2 (EU-COVAT-1-AGED): a trial conducted within the VACCELERATE network
Source: Trials. 2022 Oct 8;23:865. doi: 10.1186/s13063-022-06791-y (PMC9547672; doi:10.1186/s13063-022-06791-y)
Supplement: Supplementary file 2 — Additional file 2. [file 13063_2022_6791_MOESM2_ESM.pdf]

Universität zu Köln

Geschäftsstelle Ethikkommission • Universität zu Köln • 50931 Köln

Uniklinik Köln, Klinik und Poliklinik für Innere
Medizin I
Herrn Prof. Dr. Oliver Cornely
Kerpener Str. 62
50937 Köln

Per EKPool

Nachrichtlich per E-Mail:
Paul-Ehrlich-Institut
Bundesinstitut für Impfstoffe und biomedizinische
Arzneimittel
Paul-Ehrlich-Straße 51-59
D- 63225 Langen

Köln, 28.09.2021

Unser Zeichen:	21-1457_1-AMG-ff
EudraCT-Nr.:	2021-004526-29
Protokoll-Nr.:	uni-koeln-4602
Sponsor:	Universität zu Köln, Albertus-Magnus Platz, 50923 Köln
Mit der Antragsstellung beauftragt:	Uniklinik Köln, Klinik und Poliklinik für Innere Medizin I<br><br>Herrn Prof. Dr. Oliver Cornely<br>Kerpener Str. 62<br>50937 Köln

A multinational, phase 2, randomised, adaptive protocol to evaluate immunogenicity and reactogenicity of different COVID-19 vaccines administration in older adults (≥75) already vaccinated against SARS-COV-2

Sehr geehrter Herr Professor Cornely,

hiermit bestätigen wir, dass die u. g. Unterlagen am 27.09.2021 eingegangen sind.

Die in unserem Schreiben vom 10.09.2021 genannten **Bedingungen sind eingetreten,** sodass nunmehr **keine ethischen oder rechtlichen Bedenken** gegen die Durchführung des Vorhabens bestehen. Hierzu wünschen wir Ihnen viel Erfolg.

Medizinische Fakultät der Universität zu Köln

Geschäftsstelle der Ethikkommission

Vorsitzender
Univ.-Prof. Dr. med.
Raymond Voltz

Leitung der Geschäftsstelle
Dr. med. Guido Grass
Telefon +49 221 478 87916

Stellv. Leitung
Dipl.-Ges.-Ök. Karolina Mäder
Telefon +49 221 478 88844

Dipl.-Biol. Alice Follmann
Telefon +49 221 478 97773

Dipl.-Ges.-Ök. Christine Grimm
Telefon +49 221 478 97772

Dipl.-Ges.-Ök. Agnieszka
Hompanera Torre
Telefon +49 221 478 87488

Christin Willgrod M.A.
Telefon +49 221 478 82902

Büroleitung
Barbara Ulhardt M.A.
Telefon +49 221 478 82900
Telefax +49 221 478 82905

ek-med@uni-koeln.de
www.ek-koeln.de

Servicezeiten:
Mo. – Do. 9.00 – 16.00 Uhr
Fr. 9.00 – 12.00 Uhr
und nach Vereinbarung

Besucheradresse:
Robert-Koch-Str. 10
Gebäude 55, 2. Etage
50931 Köln

Postanschrift:
Gebäude 55, Kerpener Str. 62
50937 Köln

Bankverbindung:
Bank für Sozialwirtschaft Köln
BLZ 370 205 00
Kto.-Nr. 8 150 000
BIC BFSWDE31

Mit freundlichen Grüßen

im Auftrag
Dr. Guido Grass

Liste der eingereichten Unterlagen

1) 2021-004526-29 DE 20210915 CTA.xml vom 27.09.2021
2) EU-COVAT-1_Aged_DEU_ICF_German_001_V02_0.pdf vom 27.09.2021
3) EU-COVAT-1_Aged_DEU_ICF_German_001_V02_0_TrackChange.pdf vom 27.09.2021
4) EU-COVAT-1_Aged_EC-eku-k_2021-09-27_Ini_Con-Appr_Resp.pdf vom 27.09.2021
5) EU-COVAT-1_Aged_EC-eku-k_2021-09-27_Ini_Con-Appr_Resp.pdf vom 27.09.2021
6) EU-COVAT-1_AGED_Modul 1_EC_2021-09-15_sign.pdf vom 27.09.2021
7) EU-COVAT-1_Aged_Sub-PROTOCOL_V02_0_sign.pdf vom 27.09.2021
8) EU-COVAT-1_Aged_Sub-PROTOCOL_V02_0_TrackChange.pdf vom 27.09.2021
9) EU-COVAT_Master protocol_V02_0.pdf vom 27.09.2021
10) EU-COVAT_Master protocol_V02_0_TrackChange.pdf vom 27.09.2021
